# Integrating microRNAs into the complexity of gonadotropin signaling networks

**DOI:** 10.3389/fcell.2013.00003

**Published:** 2013-12-27

**Authors:** Kelly León, Nathalie Gallay, Anne Poupon, Eric Reiter, Rozenn Dalbies-Tran, Pascale Crepieux

**Affiliations:** ^1^BIOS Group, INRA, UMR85, Unité Physiologie de la Reproduction et des Comportements, Nouzilly, France; CNRS, UMR7247, Nouzilly, France; Université François RabelaisTours, France; ^2^BINGO Group, INRA, UMR85, Unité Physiologie de la Reproduction et des Comportements, Nouzilly, France; CNRS, UMR7247, Nouzilly, France; Université François RabelaisTours, France

**Keywords:** gonadotropins, microRNAs, G protein-coupled receptors, signaling networks, Sertoli cells, granulosa cells

## Abstract

Follicle-stimulating hormone (FSH) is a master endocrine regulator of mammalian reproductive functions. Hence, it is used to stimulate folliculogenesis in assisted reproductive technologies (ART), both in women and in breeding animals. However, the side effects that hormone administration induces in some instances jeopardize the success of ART. Similarly, the luteinizing hormone (LH) is also of paramount importance in the reproductive function because it regulates steroidogenesis and the LH surge is a pre-requisite to ovulation. Gaining knowledge as extensive as possible on gonadotropin-induced biological responses could certainly lead to precise selection of their effects *in vivo* by the use of selective agonists at the hormone receptors. Hence, over the years, numerous groups have contributed to decipher the cellular events induced by FSH and LH in their gonadal target cells. Although little is known on the effect of gonadotropins on microRNA expression so far, recent data have highlighted that a microRNA regulatory network is likely to superimpose on the signaling protein network. No doubt that this will dramatically alter our current understanding of the gonadotropin-induced signaling networks. This is the topic of this review to present this additional level of complexity within the gonadotropin signaling network, in the context of recent findings on the microRNA machinery in the gonad.

## Introduction

The role of the gonadotropins, Follicle-stimulating hormone (FSH) and luteinizing hormone (LH), in the control of reproductive function in Mammals is widely acknowledged, hence the use of their recombinant or purified surrogates to complement fertility defects in women, and to synchronize ovulation in breeding animals. The gonadotropin-induced signaling pathways have been the matter of extensive investigations, in part because a more precise knowledge of their action within the cell could help avoiding some unwanted side effects caused by their *in vivo* administration. To induce complex signaling networks leading to integrated biological responses, gonadotropins interact with their cognate G protein-coupled receptors (GPCR), expressed at the surface of somatic cells within the male and female gonad. Whereas the transcriptome alteration induced by FSH in the male and female gonad has been analyzed (McLean et al., 2002; Sasson et al., 2003; Sadate-Ngatchou et al., 2004; Meachem et al., 2005; Perlman et al., 2006), as well as the post-translational modifications of signaling effectors (Gloaguen et al., 2011), the role of post-transcriptional regulations and their putative implication in gonadotropin-induced signaling network have been underappreciated to date. Notably, the role of microRNA in regulating cell signaling induced by FSH and LH now appears as an emerging field in the control of reproductive function, at the molecular level. As microRNAs are thought to constitute a *bona fide* network, intertwined with cell signaling pathways, it is now of great interest to discuss the role that those microRNAs could potentially play in regulating gonadotropin-induced signaling within their natural target cells in the gonad. How these microRNA networks might regulate the compartmentalization of gonadotropin signaling components and might control the reaction rates of these signaling biochemical reactions will be discussed.

## MicroRNAs from the molecule to the network

The discovery of the first microRNA, *C. elegans* Lin-4, in 1993 (Lee et al., 1993; Wightman et al., 1993) has profoundly revolutionized our perception and understanding of gene regulation. At that time, small antisense RNA were tedious to identify by standard genetic approaches, but, since then, the use of next-generation sequencing and its ongoing technological improvements has pervaded the benches, leading to the identification of 1872 mature microRNAs in human, 1186 in mouse and 449 in rat, according to the Mirbase database (www.mirbase.org, release 20, June 2013).

**Figure 1:**
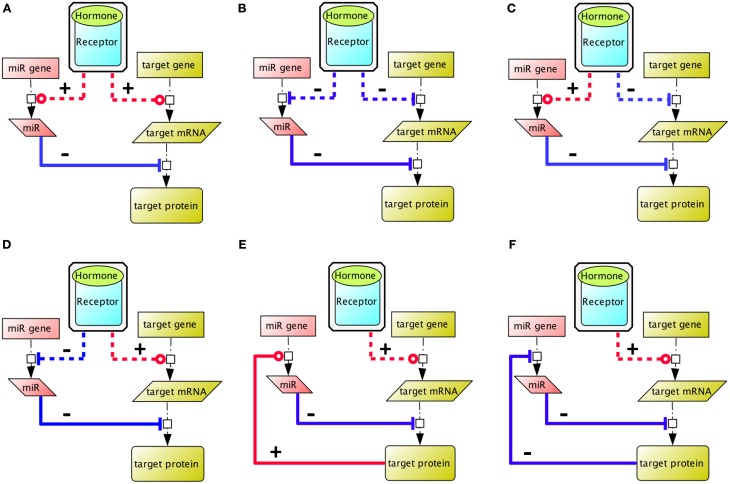
**Different ways whereby co-regulation of a microRNA circuit and gene circuit by a hormone input can impact on the global equilibrium within the ultimate expression pattern**. **(A,B)** The hormone regulates positively (red) and negatively (blue) the microRNA and the target gene expression, hence the resulting target protein expression level depends on the expression ratio of microRNA vs. target mRNA. **(C)** This box illustrates that target gene expression tends to be lower in cells where the targeting microRNA are expressed (Farh et al., 2005; Sood et al., 2006). **(D)** The hormone inhibits the expression of a microRNA, to enable gene expression of the target protein, as illustrated by the action of FSH on miR23, to stabilize PTEN expression in Sertoli cells (Nicholls et al., 2011). **(E,F)** The target gene regulates its own expression level via a negative (blue) or positive (red) feedback mechanism. Indirect reactions are figured with dashed lines. Adapted from Tsang et al. (2007).

MicroRNAs are endogenous ≃22-nucleotide long, non-coding RNAs that regulate gene expression post-transcriptionally, upon specific base-pairing of their 5′ (the seed) generally to the 3′untranslated region (UTR) of a target mRNA. They are thought to act primarily (about 80%) by destabilizing cytoplasmic mRNA (Guo et al., 2010). However, they can also regulate mRNA translation, and it has been proposed that the effect of microRNA complexes on translation oscillates between an inhibitory and a stimulating action during the cell cycle in actively cycling cells like Human Embryonic Kidney (HEK) 293 cells (Vasudevan et al., 2007). Interestingly, during physiological differentiation processes, microRNAs are considered to support mRNA cell-specificity (Farh et al., 2005; Sood et al., 2006), and overall, it is now admitted that they confer robustness to gene regulation (Cui et al., 2006; Tsang et al., 2007; Lin et al., 2013). To regulate cell fate, they exert diverse actions on signaling networks: positive feedback loops, mutual negative feedback loops, or combining positive and negative feedbacks (Figure 1) (Tsang et al., 2007).

Functional approaches by knocking-out microRNA-encoding genes have proven limited potential. That is, functional redundancy of microRNA with shared seed sequences has precluded the observation of an obvious phenotype by gene knockout of individual microRNA. Even knocking-out an entire family of microRNA sharing the same seed might be inconclusive, given that mismatches between microRNA and target are tolerated. Alternatively, the enzymes that tune microRNA processing have been knocked-out in mouse (Table 1). Consistent with a key role of microRNAs in cell-lineage decision-making, knockout of the *Dicer* gene that encodes an RNase III processing the maturation of both microRNA and endogenous short-interfering RNA, is embryonic lethal at E7.5 (Bernstein et al., 2003), similarly to knock-out of the gene encoding DiGeorge Syndrome critical region gene 8 (DGCR8) or the DGCR8 co-factor, Argonaute 2 (AGO2) (Morita et al., 2007; Wang et al., 2007).

**Table 1:** **Phenotypic consequences of the depletion of genes involved in microRNA biogenesis in mouse**.

**Gene**	**Penetrance**	**Phenotype**	**References**
Dicer	Whole-body	Embryonic lethal E7.5	Bernstein et al., 2003
DGCR8	Whole-body	Embryonic lethal E6.5	Wang et al., 2007
AGO2	Whole-body	Embryonic lethal before E7.5	Morita et al., 2007
AGO2	PGC (TNAP-Cre starts at E10)	Similar as wt	Hayashi et al., 2008
Dicer	PGC (TNAP-Cre starts at E9.5 or E10)	Elongating spermatid depletion; Spermatogenic arrest prior meiosis	Hayashi et al., 2008; Maatouk et al., 2008
Dicer	Spermatogonia (Ddx4-Cre starts at E15–E18)	Meiosis and spermiogenesis defects	Romero et al., 2011
Dicer	Spermatogonia (Ngn3-Cre starts at P5)	Spermiogenesis defects	Korhonen et al., 2011
Dicer	Spermatogonia (Stra8-Cre starts at P3)	Spermatocyte and spermatid depletion	Wu et al., 2012
Drosha	Spermatogonia (Stra8-Cre starts at P3)	Spermatocyte and spermatid depletion	Wu et al., 2012
Dicer	Sertoli/Leydig cells (SF1-Cre starts at E9.5)	Testis cords degenerate	Huang and Yao, 2010
Dicer	Sertoli cells (AMH-Cre starts at E14.5)	Meiosis and spermiogenesis defects	Papaioannou et al., 2009
*Dicer*	Oocyte (ZP3-cre starts in activated primordial follicles)	Abnormal spindle and chromosome alignment in maturing oocytes; meiotic arrest	Murchison et al., 2005; Tang et al., 2007
*Dicer*	Oocyte (ZP3-cre starts in activated primordial follicles; Aplp-cre starts in PGC)	Spindle breakdown and chromosome displacement during the metaphase to anaphase transition; meiotic arrest	Mattiske et al., 2009
*Ago2*	Oocyte (ZP3-cre starts in activated primordial follicles)	Abnormal spindle and chromosome alignment; meiotic arrest	Kaneda et al., 2009
*Dgcr8*	Oocyte (ZP3-cre starts in activated primordial follicles)	Normal oocyte maturation, normal blastocysts; reduced litter size	Suh et al., 2010
*Dicer*	Granulosa and mesenchyme-derived cells of the oviduct and uterus (Amhr2-cre starts in growing follicles)	Increased apoptosis of granulosa cells; decreased ovulation rate; decreased fertilization; normal *in vitro* but delayed and decreased *in vivo* embryo development (abnormal oviduct and uterus)	Hong et al., 2008; Nagaraja et al., 2008
*Dicer*	Granulosa (Amhr2-cre starts in growing follicles)	Larger pool of primordial follicles in neonatal ovaries, but more activated and/or degenerated follicles (oocytes) up to 8 months; abnormal luteal cells	Lei et al., 2010

## MicroRNAs in the gonad: 10 years after

In 2004, low-density human microRNA chips (Barad et al., 2004; Liu et al., 2004) have been designed and thereafter, 29 microRNAs from adult mouse testes were cloned. These seminal experiments settled the foundation for a role of microRNAs in regulating gonadal functions. By DNA electroporation of fluorescent reporter genes *in vivo*, it was elegantly demonstrated that RNA interference is active during the whole spermatogenetic process (Shoji et al., 2005), a finding further supported by high-throughput microarray analyses of microRNAs in immature vs. mature mouse whole testes (Yan et al., 2007). Later on, microRNAs were directly cloned from purified populations of spermatogenic cells (Ro et al., 2007a) and from the ovary (Ro et al., 2007b). A prominent feature of male post-meiotic, haploid germ cells is the chromatoid body, a perinuclear structure enriched in components of RNA interference silencing complex (miRISC), such as microRNAs, Dicer, Argonaute proteins and the mouse Vasa homolog (MVH) RNA helicase (Kotaja et al., 2006). This is relevant to the observation that compaction of chromatin that takes place in haploid germ cells precludes transcription, so that post-transcriptional regulation mainly occurs (Tanaka and Baba, 2005). The relative expression level of these components in pachytene spermatocytes, in round spermatids and in elongated spermatids suggests a programmed, stage-specific involvement of the different elements of the RNA interference machinery in spermatogenesis (Gonzalez-Gonzalez et al., 2008a). When the *Dicer* gene has been knocked-out specifically with tissue-nonspecific alkaline phosphatase (*TNAP*)-driven Cre recombination, spermatogenic defects were detected prior to meiosis (Hayashi et al., 2008; Maatouk et al., 2008), which seemed to be independent of AGO2 activity (Hayashi et al., 2008) (Table 1). However, in these models, the primary defects occurred in primordial germ cells (PGC), which is too early in development to figure out clearly the bases of the spermatogenesis defects observed in the adult. Therefore, *Dicer* has also been deleted in germ cells with Cre recombinase being expressed in late development (Papaioannou et al., 2009; Romero et al., 2011) and in the neonatal life (Korhonen et al., 2011; Wu et al., 2012). In these mice, the observed phenotype was consistently a defect in spermiogenesis leading to depletion in haploid cells.

Although they are endowed with microRNA-independent functions (Johanson et al., 2013), Dicer and Drosha are renowned for their implication in microRNA biogenesis. Whereas Dicer is involved in the biogenesis of both endogenous short-interfering RNAs (siRNAs) and microRNAs, the Drosha RNase III is amenable for microRNA biogenesis solely (Lee et al., 2003), and its gene has also been conditionally knocked-out in germ cells (Wu et al., 2012). The resulting phenotype is a severe impairment of the haploid phases of spermatogenesis, as a result of meiotic defects (Table 1).

In comparison, in the developing oocyte, microRNA biogenesis appears suppressed. *Dicer* knockdown by synthetic siRNA as well as oocyte-restricted knockout of *Dicer* or *Ago2* have shown that both genes are required for proper oocyte maturation before ovulation (Murchison et al., 2005; Kaneda et al., 2009; Mattiske et al., 2009; Liu et al., 2010). However, several lines of evidence have indicated that endogenous siRNAs and piwi-interacting RNAs (piRNAs), rather than microRNAs, are important for oocyte maturation and ovulation (Tam et al., 2008; Watanabe et al., 2008a,b; Ma et al., 2010; Suh et al., 2010). They include the moderate phenotype of mice with oocyte-restricted depletion of *Dgcr8*, the use of reporter constructs, and the profiling of small RNAs. Actually, efficiency of siRNAs in the oocyte has been very recently ascribed to an oocyte specific isoform of Dicer (Flemr et al., 2013). However, it cannot be excluded that microRNA could be synthesized during the earliest stages of oogenesis and stored for regulating gene expression later in the developing oocyte.

## MicroRNAs in gonadal somatic cells

The cellular targets of gonadotropins are somatic cells of the male and female gonad. Initially, model organisms have provided evidence for a role of microRNA in regulating somatic cell function. In *Drosophila* ovary and testis, the germline stem cells (GSCs) and somatic stem cells (SSCs) are housed together in a common niche. This niche provides signals that regulate GSC self-renewal and differentiation. The SSCs are non-dividing and enclose the GSC daughter cell that will ultimately give rise to sperm or eggs (Fuller and Spradling, 2007; Kirilly and Xie, 2007; Voog and Jones, 2010; de Cuevas and Matunis, 2011). Previous studies suggested the importance of microRNA biogenesis in the maintenance of ovarian stem cells. Mature microRNAs are generated from their precursors by Dicer-1 (Dcr-1) (Lee et al., 2004) and the double-stranded RNA binding protein Loquacious (Loqs) (Forstemann et al., 2005). Dcr-1 is required for GSC and SSC maintenance in *Drosophila* ovary (Jin and Xie, 2007). Similarly, *loqs* mutants fail to maintain the self-renewal of GSCs (Forstemann et al., 2005). Argonaute 1 (AGO1) also plays an essential role in the maintenance of GSCs, since overexpression of *Ago1* leads to GSC overproliferation, whereas loss of *Ago1* results in the loss of GSCs (Yang et al., 2007).

In contrast to the ovary, the requirement for microRNA pathway components in the Drosophila testis has not been determined extensively. One example is provided by miR-7, a microRNA that could play a role in GSCs maintenance by targeting the 3′ UTR of *Bag-of-marbles* (*Bam*) mRNA, which encodes a key differentiation factor (Pek et al., 2009). In addition, a novel microRNA-310/313 cluster has been identified as an antagonist of the Wingless (*Drosophila* Wnt) pathway. A crucial effector of this pathway is Armadillo (Arm), which is highly expressed in the hub cells that are in direct contact with the GSC and SSC in the testis (Yamashita et al., 2003). The microRNA-310/313 cluster can modulate Arm levels by directly targeting the 3′-UTRs of the *Arm* mRNA. Hence, as expected, microRNA-310/313-deficient flies cannot modulate Arm protein levels or activity and exhibit abnormal germ and somatic cell differentiation in the male gonad (Pancratov et al., 2013). Interestingly, the Wnt pathway is also very important in mammalian follicular development, since it fine-tunes the responsiveness to LH and FSH gonadotropins (Fan et al., 2010).

In Drosophila, the dependency of the niche on gonadotropin-like hormones is unlikely, and the only homolog hormones, Glycoprotein Hormone α2 (GPA2) and Glycoprotein Hormone β 5 (GPB5), have recently been proposed to be antidiuretic hormones, and probably not *bona fide* gonadotrope hormones (Sellami et al., 2011).

In Mammals, microRNAs have also been detected in mouse Sertoli cells (Gonzalez-Gonzalez et al., 2008b), in a structure resembling the nucleolus (Marcon et al., 2008). Their importance in gonadal physiology has been demonstrated by Sertoli cell-selective knock-out of the *Dicer* gene in mouse, which has unraveled the role of microRNA in supporting cell lineage of the testis, by regulating the expression of genes essential for meiosis and spermiogenesis (Papaioannou et al., 2009). Sertoli cell development itself was impaired as soon as P5, exhibiting major alterations of the testis proteome (Papaioannou et al., 2011). Consequently, Sertoli-cell restricted *Dicer* knocked-out mice are infertile. In addition, when achieved as early as E9.5, Sertoli/Leydig-cell specific disruption of *Dicer* severely compromises testis cord evolution (Huang and Yao, 2010). In the ovary, gonadotropins control the terminal phase of folliculogenesis starting when granulosa cells express the FSH receptor. Influence of canonical microRNA on ovarian function appears to be primarily through somatic cells. Indeed, selective disruption of Dicer expression in granulosa cells altered ovarian activity resulting in a decreased ovulation rate (Hong et al., 2008; Nagaraja et al., 2008; Gonzalez and Behringer, 2009; Lei et al., 2010). FSH affects the expression of a range of microRNA in cultured rat granulosa cells, with putative target genes involved in multiple signaling pathways (Yao et al., 2009, 2010). The challenge is now to identify the physiologically relevant targets.

## FSH signaling properties: the bright side of the moon

Before integrating microRNAs into the complexity of FSH-induced signaling network, here is a brief overview on how this signaling network is organized. In Mammals, FSH ability to alter gene transcription in granulosa and Sertoli cells has long been recognized (McLean et al., 2002; Sasson et al., 2003; Sadate-Ngatchou et al., 2004; Meachem et al., 2005; Perlman et al., 2006). These effects are conveyed from the plasma membrane to the nucleus through the Gαs/cAMP/protein kinase A (PKA) signaling cascade essentially (Means et al., 1974; Dattatreyamurty et al., 1987). More precisely, Gαs-coupling leads to cAMP accumulation that in turn activates PKA (Zeleznik et al., 2003; Escamilla-Hernandez et al., 2008) and Exchange Protein directly Activated by cAMP (EPAC) (Gonzalez-Robayna et al., 2000; Wayne et al., 2007). Amongst other cytosolic targets, PKA has been reported to trigger p38 (Maizels et al., 1998), Extracellular signal-Regulated Kinases (ERK) 1,2 (Cottom et al., 2003) and p70 ribosomal S6 Kinase (p70S6K) (Lécureuil et al., 2005) whereas EPAC has been associated with p38 and Akt activation (Wayne et al., 2007; Choi et al., 2009). Gαi-coupling counteracts cAMP accumulation but can also lead to ERK1,2 activation (Crépieux et al., 2001).

Over the last decade, this picture has become considerably more complex as activated the FSH receptor (FSHR) has been reported to couple to multiple other transduction mechanisms ultimately leading to the activation of an intricate intracellular network (Gloaguen et al., 2011; Ulloa-Aguirre et al., 2011). Importantly, this FSH-induced signaling network is now perceived as regulating protein translation (Musnier et al., 2010, 2012) and as controlling the fate of FSH-targeted cells (Loss et al., 2007; Richards and Pangas, 2010) in addition to its effects at the level of gene transcription.

Several Gαs-independent mechanisms capable of functionally coupling the FSHR with intracellular signaling cascades have been reported to physically and/or functionally interact with the FSHR and to trigger downstream signaling events (Zeleznik et al., 2003; Escamilla-Hernandez et al., 2008; Gloaguen et al., 2011). These mechanisms involve Gαi (Arey et al., 1997; Crépieux et al., 2001), β-arrestins (Kara et al., 2006; Wehbi et al., 2010a,b; Tranchant et al., 2011), Epithelial Growth Factor Receptor (EGFR) (Cottom et al., 2003; Andric and Ascoli, 2006; Yang and Roy, 2006; Wayne et al., 2007) and Adaptor protein, phosphotyrosine interaction, PH domain and leucine zipper containing 1 (APPL1) (Nechamen et al., 2004, 2007; Thomas et al., 2011). Although the coupling mechanisms that promote Phosphoinositide 3-kinase (PI3K) (Zeleznik et al., 2003) and Phosphatase and Tensin homolog deleted on chromosome 10 (PTEN) (Dupont et al., 2010) activation upon FSH exposure are as yet unidentified, APPL1 is a likely candidate because it has been reported to trigger downstream signaling through PI3K (Nechamen et al., 2004). Besides, the interaction between APPL1 and the FSHR has been shown to be necessary for the activation of inositol-phosphate/calcium pathways upon FSH exposure (Thomas et al., 2011) of the KGN granulosa cell line. All of these coupling mechanisms subsequently connect to second messenger-activated effectors and/or to downstream signaling cascades.

Like most GPCRs, the activated FSHR has been shown to specifically interact with G protein-coupled receptor kinases (GRKs) and with β-arrestins (Reiter and Lefkowitz, 2006). Initially, these proteins have been related to the control of desensitization, internalization and recycling of the receptor but, more recently, β-arrestins have been shown to act as G protein-independent signal transducers at the FSHR, leading to the activation of ERK1,2 and rpS6, in heterologous cell lines (Kara et al., 2006; Wehbi et al., 2010a,b; Tranchant et al., 2011). More generally, they enable post-translational modifications of a wide array of intracellular targets (Xiao et al., 2010), by acting as multifunctional scaffolds of multiple protein partners (Xiao et al., 2007). Hence, β-arrestins are likely involved in the activation of numerous other signaling pathways at the FSHR, in FSH natural target cells, although this remains to be demonstrated experimentally.

Transduction of FSH signal within granulosa cells also leads to transactivation of the epithelial growth factor receptor, by autophosphorylation of the EGFR (Cottom et al., 2003; Wayne et al., 2007; Zhang et al., 2007). In addition, when the EGFR is inhibited, a decrease in the ability of FSH to induce ERK and Cyclin-dependent kinase (CDK) 4 activation (Cottom et al., 2003; Andric and Ascoli, 2006; Yang and Roy, 2006; Wayne et al., 2007) or PI3K, Akt, (McDonald et al., 2006; Wayne et al., 2007) and AMP-activated protein kinase (AMPK) (Kayampilly and Menon, 2009)/Glycogen Synthase Kinase (GSK) 3β (Alam et al., 2004, 2009; Fan et al., 2010) has been reported in granulosa cells.

## Integrating microRNA regulation in gonadotropin signaling network

**Figure 2:**
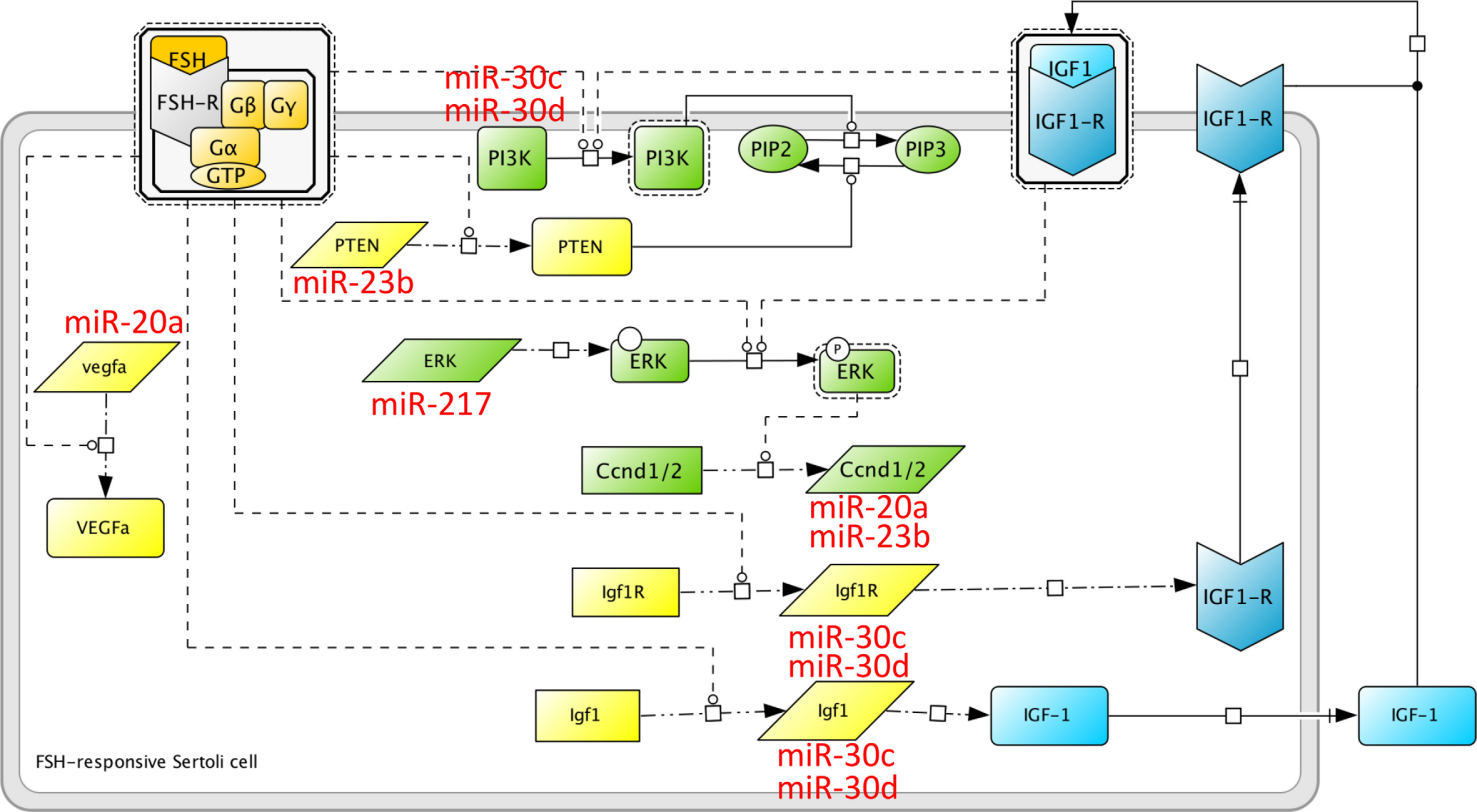
**MicroRNA induced by FSH at spermiogenesis**. A microRNA network superimposes on the protein network induced by FSH. Here are shown some microRNAs whose expression is altered upon *in vivo* FSH suppression, i.e., miR-23b, miR-30c and miR-30d, mir-20a and miR-217, as reported in Nicholls et al. (2011). miR-30c and miR-30d target the IGF-1 system, as well as the PI3K pathway. According to Khan et al. (2002), PI3K is activated directly by FSH in the prepubertal rat, and indirectly, through the IGF1-R, in the neonate. miR-23b could impair the post-transcriptional regulation of PTEN induced by FSH (Dupont et al., 2010), and could potentially target Cyclin D1. Whether FSH regulates Cyclin D1 at the transcriptional or post-transcriptional level is not known yet.

Based on the importance of microRNAs in the male gonad and in Sertoli cells more specifically, as presented above, it can be anticipated that a complex microRNA network will superimpose intricately with this complex FSH signaling network, to determine the ultimate cell response to the hormone. The first large-scale work exploring this assumption has focused on the molecular bases of spermiation, in a rat model where FSH and testosterone action was suppressed *in vivo* (Nicholls et al., 2011). At this stage of spermatogenesis, which is particularly sensitive to hormone regulation, elongated spermatids are released away from Sertoli cells, following complex membrane rearrangements. Therefore, this study has focused on signaling mechanisms related to Sertoli cells/germ cells adhesion. By probing a microRNA micro-array, it appeared that 163 microRNAs were responsive to hormone treatment in *in vitro* Sertoli cells cultured from P20 rats. One of the mRNA candidate targets that came out from this analysis was the *pten* mRNA (Figure 2) whose 3′ region exhibits putative target sites for miR-23b and miR-217. Individual miR efficiency being weak, it is common that several distinct miR act in concert on a single mRNA, to optimize repression. The hormonal input would lead to degradation or synthesis inhibition of the *pten*-directed microRNA, resulting in *pten* mRNA stabilization and protein accumulation in the apical region of the cells, in the vicinity of mature spermatids, possibly to regulate cell adhesion (Nicholls et al., 2011). These data illustrate another property of microRNAs, in the compartmentalization of mRNA to be translated, as previously shown for locally translated transcripts in neurons (Kim et al., 2004).

Interestingly, our group had previously reported that the PTEN protein level was massively enhanced (about 80-fold above basal in average, at maximum) following *in vitro* cell stimulation by FSH, leading Sertoli cells to achieve terminal differentiation (Dupont et al., 2010). Given that FSH enhanced PTEN protein level within minutes, we assumed that this regulation occurred post-transcriptionally, in agreement with Stanton's group (Nicholls et al., 2011). However, it appeared that recruitment of the *pten* mRNA into the polysomes was not increased upon FSH stimulation (Musnier et al., 2012). Therefore, we assume that the microRNA targeting the *pten* mRNA might inhibit the elongation phase of translation, rather than its recruitment into the polysomes. Previous evidence that microRNA could associate to polysomes and block the elongation phase of translation have been provided (Maroney et al., 2006). Nevertheless, the corresponding mechanisms are still unclear (Fabian et al., 2010), and the most recent views now favor an implication of microRNA in translation initiation rather than elongation (Ricci et al., 2013). In contrast to *pten*, recruitment of the *vegfa* mRNA to the polysomes is enhanced upon FSH stimulation in Sertoli cells from prepubertal rats (Musnier et al., 2012). The *vegf* mRNA is a putative target of miR-20a, whose expression is stimulated by FSH at spermiogenesis. Hence, in this case, miR-20a might impact at the level of *vegfa* mRNA translation initiation.

In addition, Stanton's group bioinformatics analysis also revealed the existence of microRNA targeting components of the ERK pathway that would be repressed in the presence of hormone. This observation, obtained from mature rats, contrasts with our finding that, in pre-pubertal rats, the abundance of ERK MAP kinase is unchanged in FSH-stimulated cells, whereas the phosphorylation level varies (Crépieux et al., 2001).

An important pathway that supports both the mitogenic phase and the nurse cell function of Sertoli cells is the Insulin-like Growth Factor 1 (IGF1)/IGF1 receptor (IGF1-R) system. IGF-I stimulates Sertoli cell proliferation *in vitro* (Borland et al., 1984) and *in vivo* ablation of the IGF system (both IGF1-R and Insulin Receptor knock-out) have convincingly entrenched its requirement for FSH-mediated mitogenic action in the pre-pubertal mouse (Pitetti et al., 2013). Based on previous observations, it is admitted that both FSH and IGF-I act on the PI3-K (Froment et al., 2007) and ERK (Crépieux et al., 2001) pathways, both involved in cell proliferation and survival. From Stanton group's analysis, miR-30c and miR-30d appear to be responsive to FSH. These microRNA potentially recognize the IGF1, IGF1-R, and PI3K mRNA (Figure 2), suggesting that the expression of these key effectors of FSH biological action is tightly regulated. In addition, FSH and IGF-I directly impact on cell cycle regulators. For example, both FSH and IGF-I can stimulate the expression of the D1 and D2 cyclins (Crépieux et al., 2001; Pitetti et al., 2013), instrumental in mediating progression through the G1 phase of the cell cycle (Figure 2). In addition to the *pten* mRNA, miR-23b could also regulate the *Cyclin D1* mRNA (Nicholls et al., 2011). We had shown previously that FSH enhanced the level of Cyclin D1 expression in Sertoli cells from neonate rats, but not from pre-pubertal rats (Crépieux et al., 2001). Therefore, it is conceivable that later on, when spermatogenesis has started, and when Sertoli cell proliferation has ceased, cyclin D1 has to be maintained at a low level within the cell. Furthermore, IGF-I inhibits the transcription of two cell cycle inhibitors, namely the *p*15^*Ink*4^ gene (Pitetti et al., 2013) and presumably the *p*21^*Cip*^ gene (Marx, 1993), via p53 dephosphorylation (Froment et al., 2007) in Sertoli cells.

Later in life, once spermatogenesis is efficient, the FSH-R- and the IGF1-R-transduced signaling share the PI3-K as a common effector to stimulate lactate production in the neonate (Khan et al., 2002), whereas FSH stimulates the PI3-K pathway independently of IGF-I before puberty (Meroni et al., 2004). Lactate production is a key nurturing function of Sertoli cells, which metabolize it from glucose that post-meiotic germ cells can then utilize as an energy source. The role of FSH in this process is first to accelerate the transport of glucose within Sertoli cells, presumably through Glucose Transporter 1 (GLUT-1) (Galardo et al., 2008), and second to enhance the activity of lactate dehydrogenase, the enzyme that converts pyruvate to lactate at the end of the metabolic chain.

## Insights onto the regulation of LH action by microRNAs

Most functional studies of microRNAs in granulosa cells have focused on the pre-ovulatory surge of LH, a critical period of the ovarian cycle. Several microRNA including miR-21, miR-122, miR-132, miR-212, and miR-136-3p are upregulated following administration of an ovulatory dose of human Choriogonadotropin (hCG) and/or supplementation of granulosa cell culture medium with the hormone (Fiedler et al., 2008; Carletti et al., 2010; Kitahara et al., 2013; Menon et al., 2013). In a functional approach, inhibiting miR-21 or miR-122 affected granulosa cell survival/apoptosis, proliferation or transition to luteinization, supporting a physiological role (Carletti et al., 2010; Menon et al., 2013). Numerous putative targets were predicted using bioinformatics. C-terminal binding protein-1 (CtBP-1), a cofactor of Steroidogenic Factor 1 (SF-1) and co-repressor of nuclear receptors, was actually regulated when blocking miR-132 and miR-212 (Fiedler et al., 2008). Finally, miR-136-3p was shown to be involved in destabilizing the LH receptor (LHR) mRNA, contributing to its down-regulation after the LH surge (Kitahara et al., 2013). Within the ovulatory follicle, a remarkable event is the expansion of the oocyte-surrounding cumulus, which changes from a compact cell mass into a dispersed structure of cells, under the action of several proteins including Pentraxin3. miR-224 was shown to function as a negative regulator of cumulus expansion *in vitro* and ovulation *in vivo* by directly targeting Pentraxin3 mRNA, consistent with miR-224 decrease in both granulosa and cumulus cells of hCG-injected immature mice after equine CG treatment (Yao et al., 2013).

In the testis also, Lin28b, an RNA-binding protein that is involved in the biosynthesis of the let-7 microRNA, is expressed in interstitial Leydig cells and its expression might be upregulated by LH/CG (Gaytan et al., 2013). Synthesis of the LH β subunit in the pituitary is itself controlled by miR-200b and miR-429, whose knock-out in mouse leads to anovulation (Hasuwa et al., 2013).

## Future directions

Spatial restriction of signaling effectors upon Sertoli cell morphological changes could correlate to differentiation, as suggested by the spatial restriction of FSH-dependent expression of PTEN to the apical region of Sertoli cells (Nicholls et al., 2011). FSH biological function shifts between 9 and 18 days *post-partum*, prior to the first wave of spermatogenesis in rodents, from first sustaining Sertoli cell proliferation to a role in metabolic and architectural support for germ cells. Not only the gene expression pattern but also the signaling pathways vary as a function of Sertoli cell post-developmental stage. Defining the molecular bases of this switch has been the matter of an intensive quest, but many possibilities still remain. An appealing explanation would be a systemic change in the microRNA network, reminiscent of the ongoing hypotheses on the maternal/zygotic transition (Walser and Lipshitz, 2011). This possibility is supported by the fact that, in the course of cell differentiation, microRNAs are considered to consolidate the transcriptional gene expression program by repressing leaky transcripts that were specific of the anterior, less differentiated stage (Stark et al., 2005). By these means they would support tissue identity (Farh et al., 2005; Sood et al., 2006). A genome-wide analysis of FSH-regulated microRNAs at different Sertoli cell developmental stages could probably address this option. With more than 60% of protein-coding genes being computationally predicted as microRNA targets (Friedman et al., 2009), bioinformatics analyses at the systems level should soon identify potential microRNA target sites in gonadotropin-induced transcriptome/translatome, with an unprecedented throughput. This will allow taking into account the microRNA regulation level in systems biology of gonadotropin signaling networks.

Beyond their physiological role, gonadotropin biological action can be hijacked in pathological disturbances such as cancer or infertility. For example, FSH can enhance ovarian cancer cells proliferation through the PI3K/Akt signal pathway (Choi et al., 2002). Induction of Vascular Endothelial Growth Factor, Cyclooxygenase 2 and survivin expression by FSH in such cells was recently reported to involve miR-27a (Lai et al., 2013). On the other hand, the screening of a high density Ovarian Cancer Disease Specific Array with human ovarian carcinoma cell mRNA suggests that LH may reduce cancer cell proliferation *via* some regulatory microRNAs (Cui et al., 2011a,b).

It has been proposed that some cellular mRNA act as *bona fide* microRNA target in one cell type or in defined physiological conditions, but as a microRNA-sequestering molecule in other cell type or conditions (Seitz, 2009). For example, endogenous transcripts named “competitive endogenous RNAs” (ceRNAs) appear to act as natural microRNA decoys (Tay et al., 2011). One of the major *conundrums* that preclude reaching the real dynamics of interactions between all of these actors is the quantitative determination of the relative ratio of microRNAs vs. ceRNA within the cell. Notably, the *pten* mRNA bears such a “non-coding” function. So far, this dimension is still out of reach regarding the regulation of gonadotropin signaling network, but no doubt that a comprehensive view of these networks will have to integrate these multidimensional regulations.

### Conflict of interest statement

The authors declare that the research was conducted in the absence of any commercial or financial relationships that could be construed as a potential conflict of interest.
